# Supplementary figures and images for: Complementary Roles of Structure and Variant Effect Predictors in RyR1 Clinical Interpretation
Source: Hum Mutat. 2025 Oct 3;2025:1834898. doi: 10.1155/humu/1834898 (PMC12513790; doi:10.1155/humu/1834898)

## MH - Malignant Hyperthermia

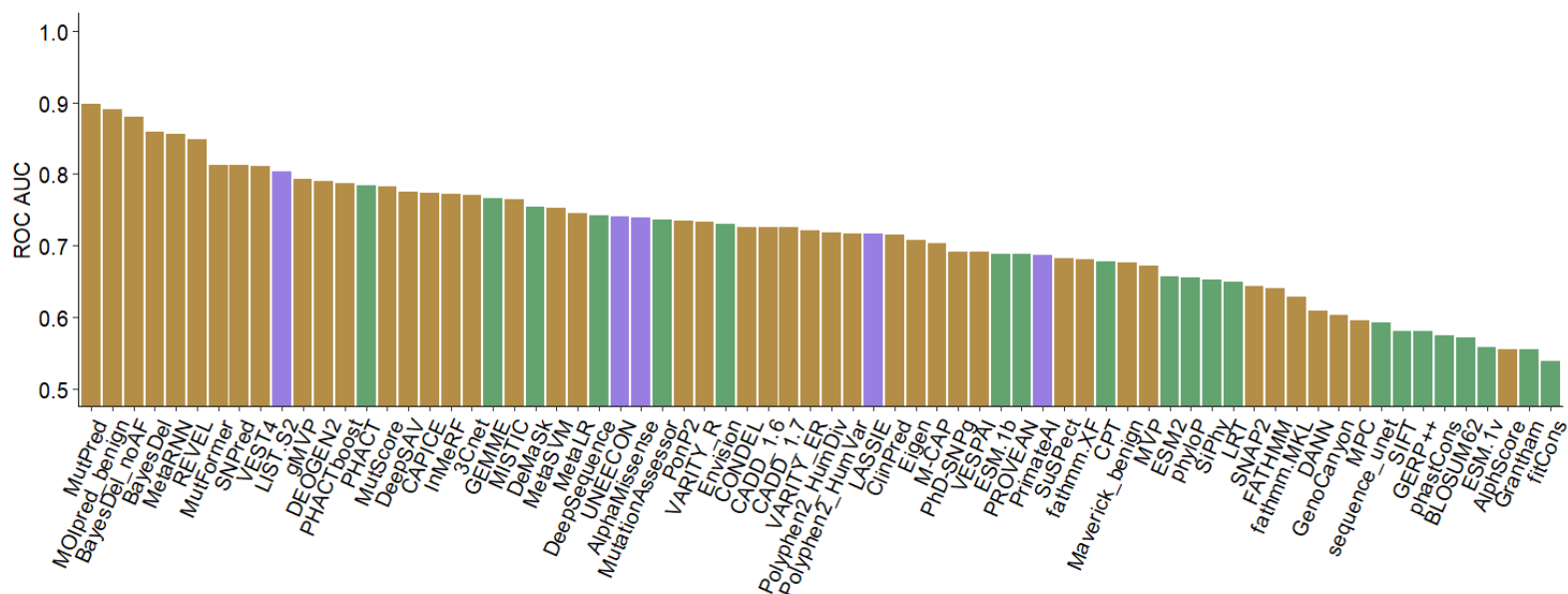

## CCD - Central Core Disease

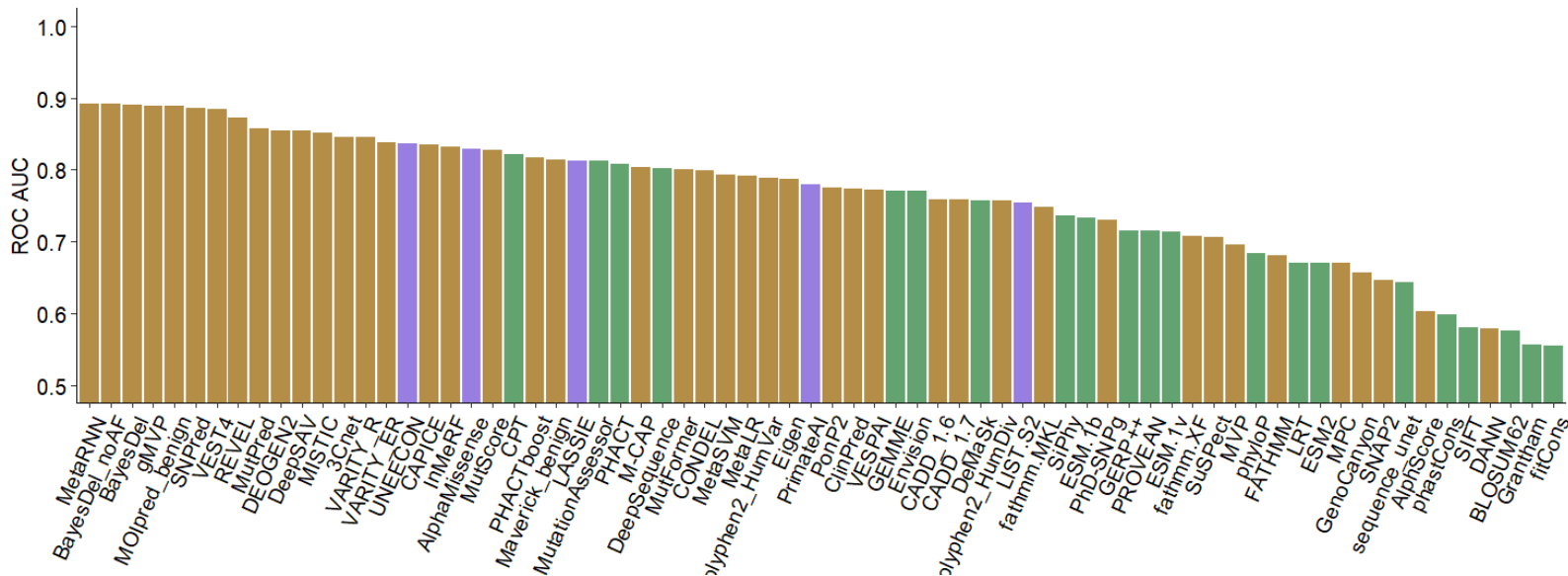

## RRD - Other dominant RYR1-related Disease

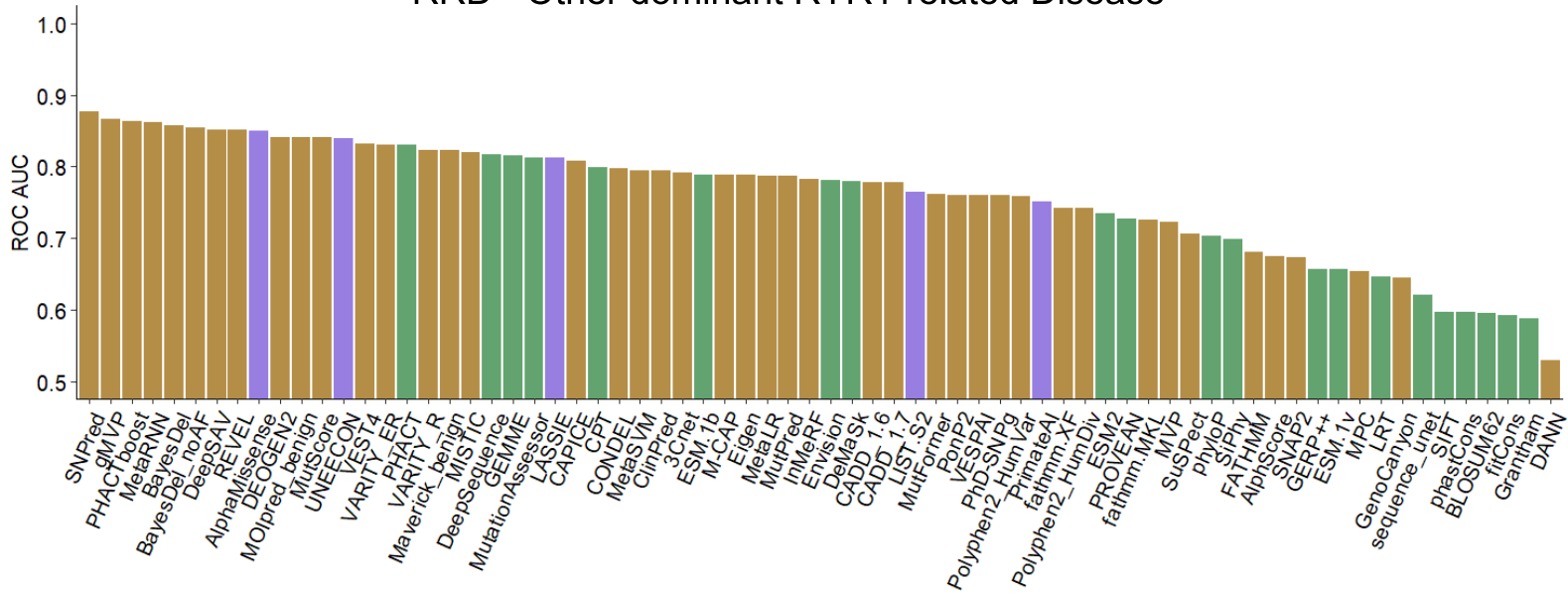

Supplement: Supporting Information 1 — Figure S1: VEP performance for the identification of pathogenic RyR1 variants by phenotypic group. Bar plot showing the performance using ROC AUC (area under the receiver operating characteristic curve) for all the VEPs used in this study when evaluating pathogenicity of variants by the MH (malignant hyperthermia), CCD (central core disease) and RRD (other dominant RYR1-related disease) groups when compared to putatively benign variants. VEPs are coloured by class: clinically trained (brown), population-free (green) and population-tuned (violet). [file 1834898.f1.pdf]

phenotype MH CCD RRD

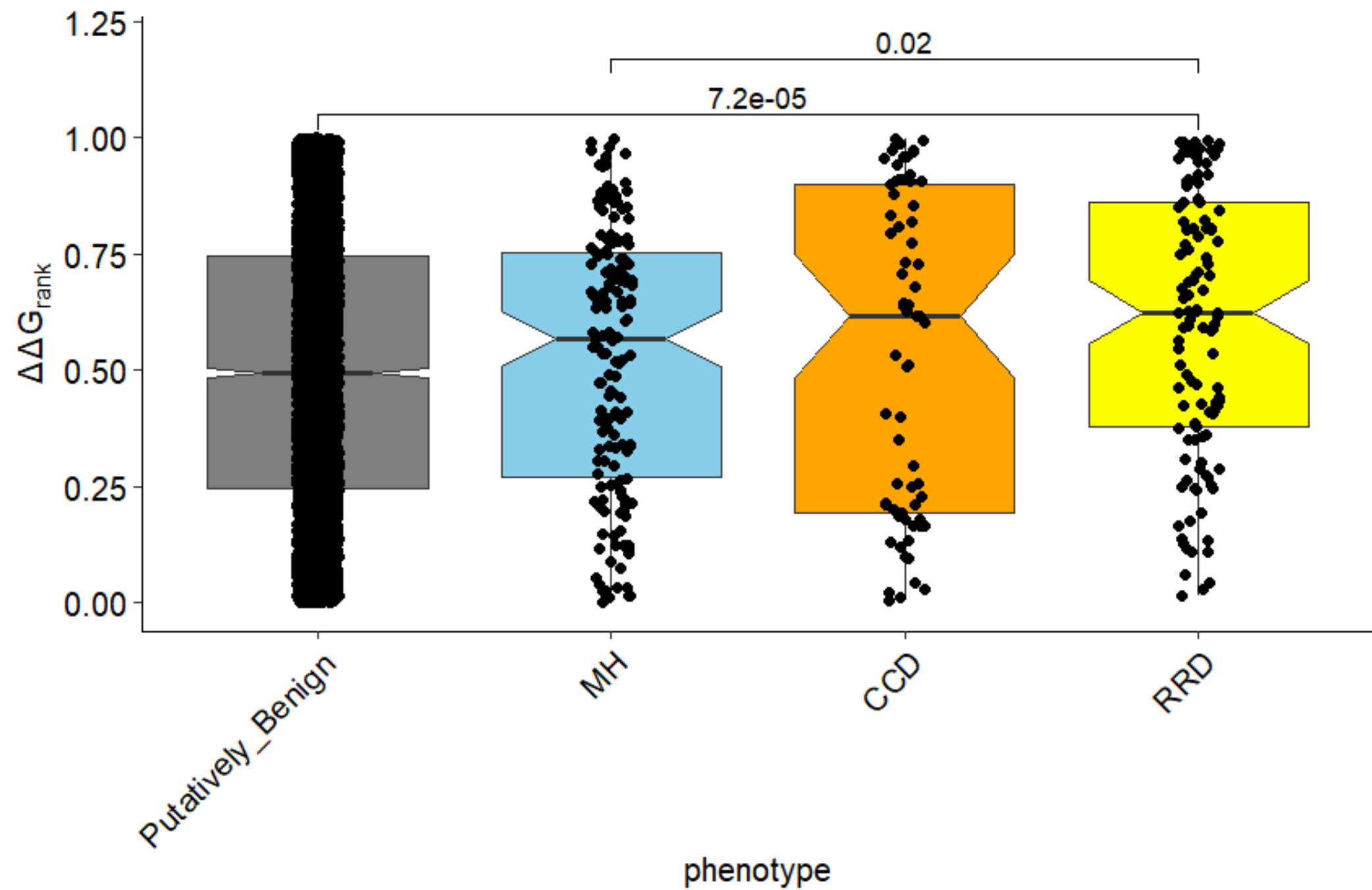

Supplement: Supporting Information 2 — Figure S2: Comparison of ΔΔGrank values from missense variants associated with different RYR1 phenotypic groups. ΔΔGrank is a recently introduced rank-normalised representation of |ΔΔG| that improves visual comparisons. [file 1834898.f2.pdf]

(A)

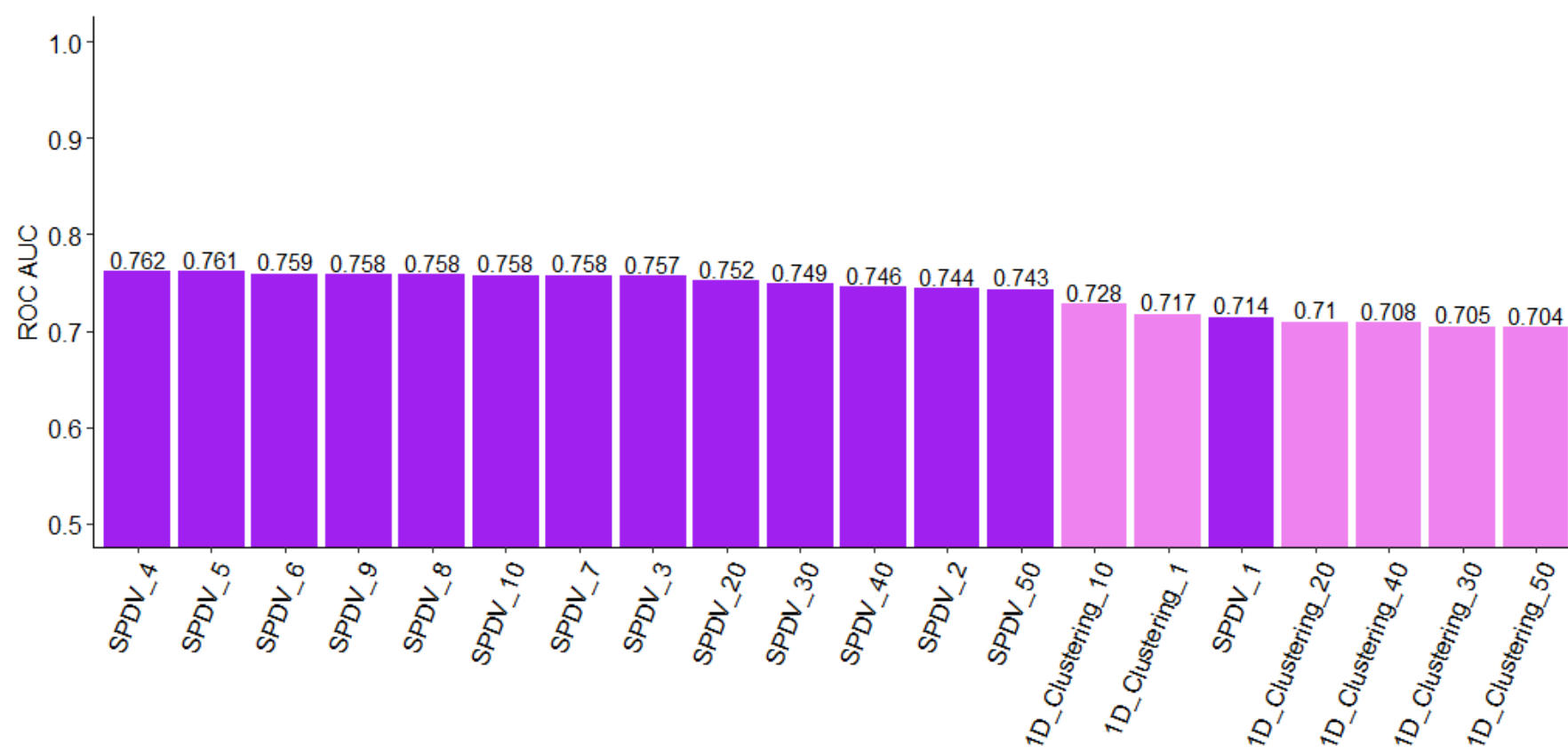

(B)

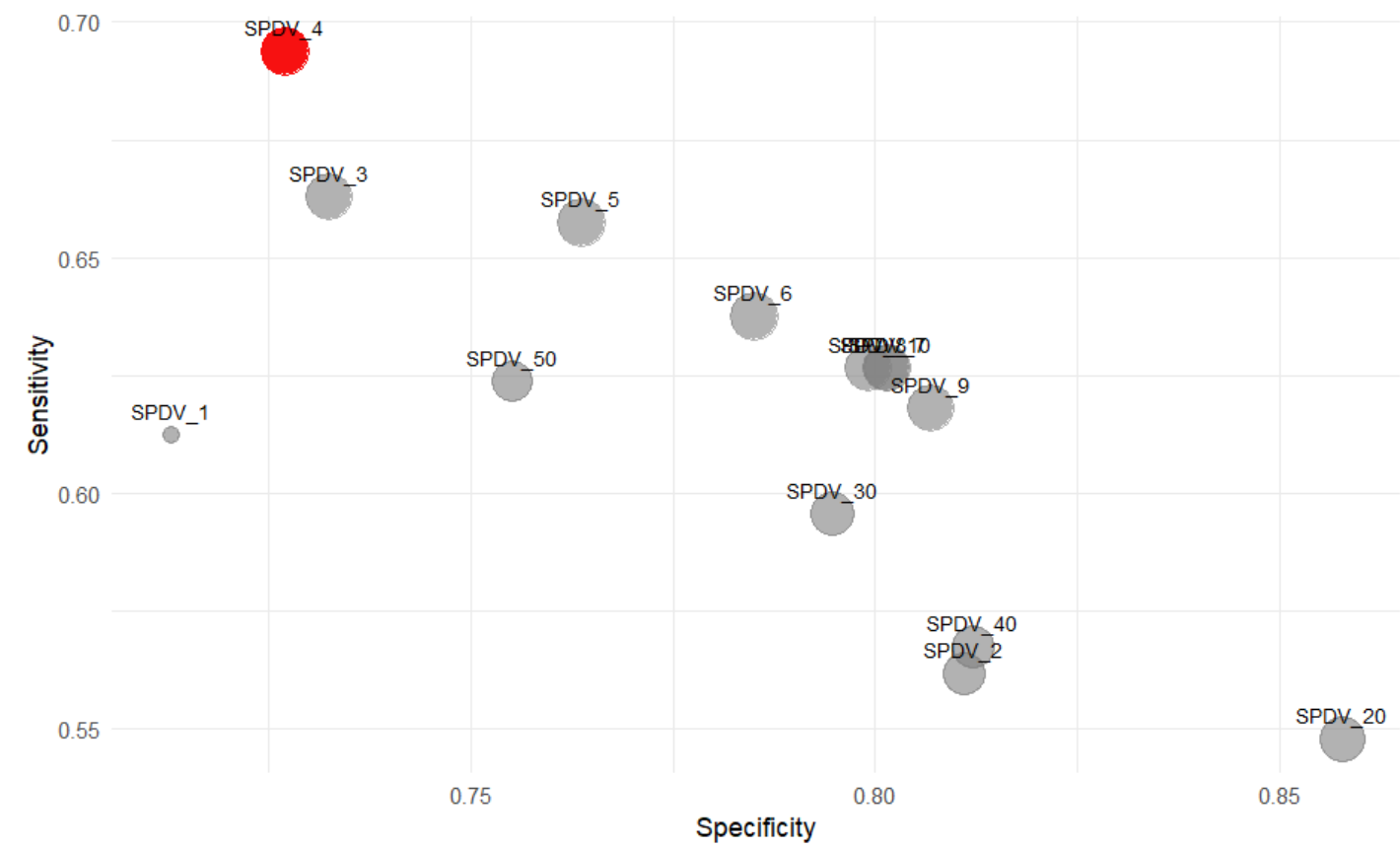

Supplement: Supporting Information 3 — Figure S3: SPDV_4 is the top-performing SPDV instance. (a) ROC AUC performance of different SPDV instances calculated using the 3D protein structure and the mean distance to the K-nearest pathogenic variants, where K = 1–10, 20, 30, 40 and 50. While performance is relatively consistent across values of K, SPDV_4 achieves the highest AUC. (b) Sensitivity versus specificity for the same set of SPDV instances, evaluated at the optimal threshold determined by the Youden index (J statistic). SPDV_4 shows the best balance between sensitivity and specificity, further supporting its selection for downstream analysis. [file 1834898.f3.pdf]

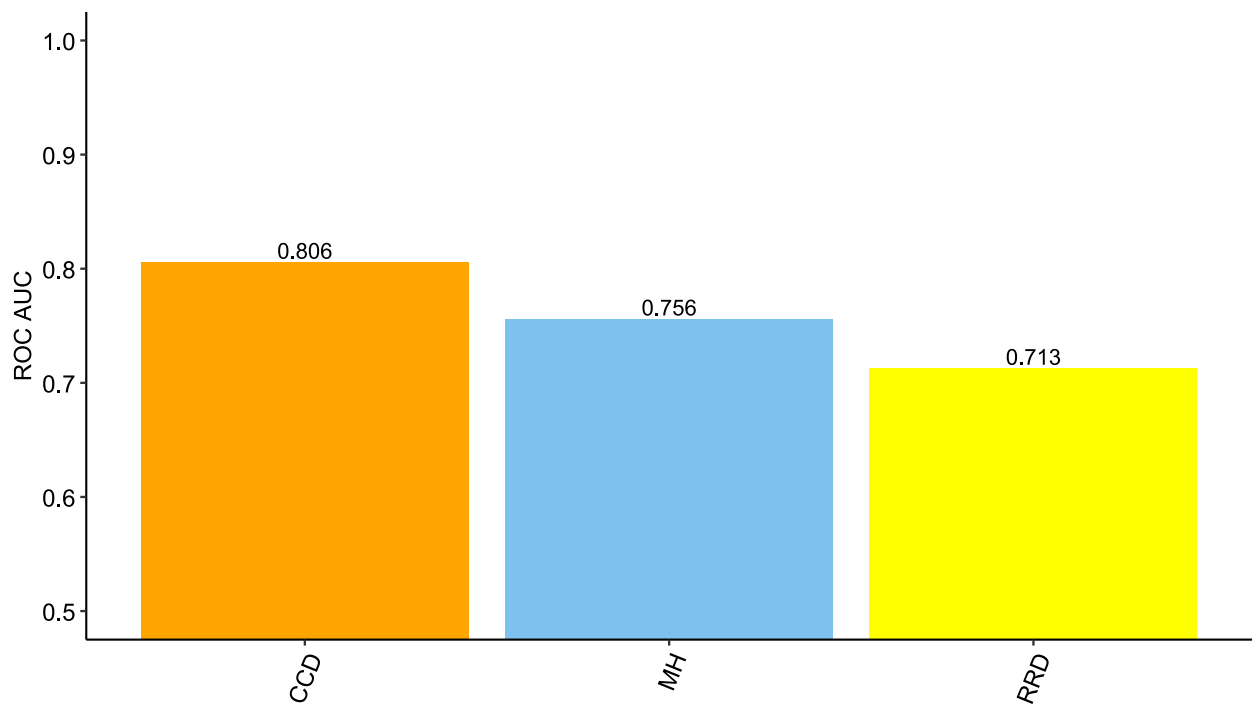

Supplement: Supporting Information 4 — Figure S4: Performance of the top-performing SPDV instances by phenotypic group. Predictive performance improves for the CCD model compared to treating all disease mutations collectively, with a top ROC AUC of 0.806 (K = 2). The MH model achieves very similar performance to SPDV calculated using the whole dataset, with a top ROC AUC of 0.760 (K = 4). The RRD model performs slightly worse with a top ROC AUC of 0.713 (K = 1). [file 1834898.f4.pdf]
